# Synthesis and biological evaluation of benzimidazole-linked 1,2,3-triazole congeners as agents

**DOI:** 10.1186/s13588-014-0014-x

**Published:** 2014-12-02

**Authors:** Karna Ji Harkala, Laxminarayana Eppakayala, Thirumala Chary Maringanti

**Affiliations:** 1 Department of Physics and Chemistry, Mahatma Gandhi Institute of Technology, Chaitanya Bharathi, Gandipet, Hyderabad, 500075 India; 2 Department of Chemistry, College of Engineering, Jawaharlal Nehru Technological University, Hyderabad, Nachupally, Karimnagar, 505501 India

**Keywords:** Benzimidazole, Clic reaction, Cytotoxicity

## Abstract

**Background:**

Benzimidazoles and triazoles are useful structures for research and development of new pharmaceutical molecules and have received much attention in the last decade because of their highly potent medicinal activities.

**Findings:**

A simple and efficient synthesis of triazole was carried out by treatment of 2-(4-azidophenyl)-1H-benzo[d]imidazole (**6**) with different types of terminal alkynes in t-BuOH/H_2_O, sodium ascorbate, and Zn(OTf)_2_, screened for cytotoxicity assay and achieved good results. A series of new benzimidazole-linked 1,2,3-triazole (**8a-i**) congeners were synthesized through cyclization of terminal alkynes and azide. These synthesized congeners **8a-i** were evaluated for their cytotoxicity against five human cancer cell lines. These benzimidazole-linked 1,2,3-triazole derivatives have shown promising activity with IC_50_ values ranging from 0.1 to 43 μM. Among them, the compounds (**8a**, **8b**, **8c**, and **8e**) showed comparable cytotoxicity with adriamycin control drug.

**Conclusions:**

In conclusion, we have developed a simple, convenient, and an efficient convergent approach for the synthesis of benzimidazole-linked 1,2,3-triazole congeners as agents.

**Graphical Abstract:**
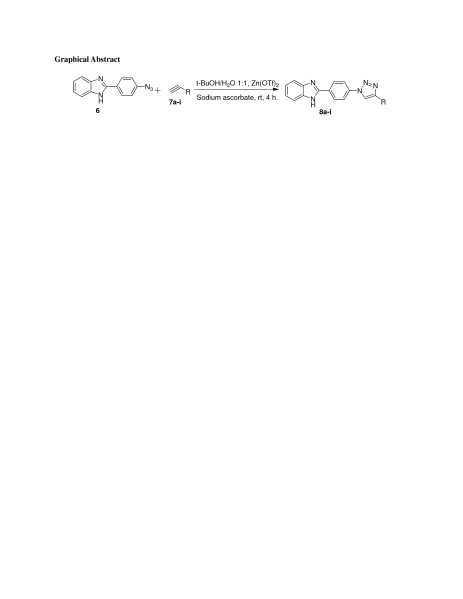
Synthesis of 1,2,3-triazole derivatives

**Electronic supplementary material:**

The online version of this article (doi:10.1186/s13588-014-0014-x) contains supplementary material, which is available to authorized users.

## Findings

**Figure 1:**
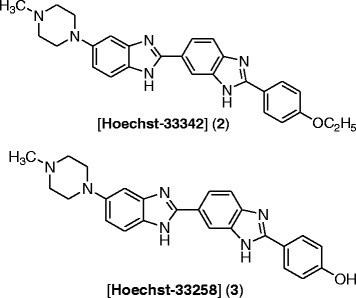
**Biologically active benzimidazole derivatives.**

Cancer is one of the terrible diseases which cause uncontrolled growth of group of cells. It remains a mean threat to human beings and cause death [1],[2]. Recently, many researchers developed safe and effective ways of treating this disease and to search for novel chemotherapeutic agents. The benzimidazole nucleus is an important pharmacophore in medicinal chemistry. The synthesis of novel benzimidazole derivatives remains a main focus of modern drug discovery. The versatility of new generation benzimidazole would represent a fruitful pharmacophore for further development of better medicinal agents. Many researchers have been attracted to benzimidazole derivatives because of their wide range of biological activity. Over the past years, there is a considerable interest in the development and pharmacology of benzimidazole. They are of wide interest because of their diverse biological activity and clinical applications [3]. Some of benzimidazole derivatives have also exhibit antimicrobial [4],[5], antitumor [6], anti-inflammatory [7], antihypertensive [8], and antiviral [9] activities. Benzimidazole moiety can also be extracted from naturally occurring compounds such as vitamin B_12_ and its derivatives, and it is similar to the structure of purins. Pyrrolo[1,2-a]benzimidazoles represent a new class of antitumour agent exhibiting cytotoxic activity against a variety of cancer cell lines [10]. Benzimidazole containing anticancer agent, [Hoechst-33342], 2′-(4-ethoxyphenyl)-5-(4-methyl-1-piperazinyl)-2,5′-bis-1H-benzimidazole (**1**), has been reported as inhibitor of topoisomerase-I [11],[12]. The other derivative of benzimidazole [Hoechst-33258] (**2** Figure 1) [13],[14] shows both *in vitro* antitumour activity, as inhibitor of DNA topoisomerase-I [15]. Hoechst 33258 a fluorescent reagent and as initially found to be active against L1210 murine leukemia. During phase I trial in humans, some responses were seen in pancreatic cancer. However, a subsequent phase II trial did not show any objective responses.

In addition, triazoles also display wide spectrum of biological activities and are widely employed as pharmaceuticals and agrochemicals. Triazoles are reported to possess antibacterial, antifungal, and antihelminthic activities [16]-[21]. They have been regarded as an interesting unit in terms of biological activity [22],[23], and some of them have also shown significant anticancer activity in many of the human cell lines [24].

In view of the biological importance of benzimidazole and 1,2,3-triazoles, to know the combined effect of both benzimidazole and 1,2,3-triazole moieties, it was considered worthwhile to synthesize certain new chemical entities having benzimidazole and 1,2,3-triazole pharmacophores in a single molecular framework, and here we have used Zn(OTf)_2_ catalyst instead of CuSO_4_. All of these congeners have been evaluated for their anticancer activity against a panel of five human cancer cell lines (Figure 1).

### Experimental section

All chemicals and reagents were obtained from Aldrich (Sigma-Aldrich, St. Louis, MO, USA) and Lancaster (Alfa Aesar, Johnson Matthey Company, Ward Hill, MA, USA) and were used without further purification. Reactions were monitored by TLC and performed on silica gel glass plates containing 60 F-254, and visualization on TLC was achieved by UV light or iodine indicator. ^1^H and ^13^C NMR spectra were recorded on Gemini Varian-VXR-unity (Palo Alto, California) (300 and 100 MHz) instrument. Chemical shifts (d) are reported in ppm downfield from internal TMS standard. ESI spectra were recorded on Micromass, Quattro LC (McKinley Scientific, Sparta, NJ, USA) using ESI + software with capillary voltage 3.98 kV and ESI mode positive ion trap detector. Melting points were determined with an electrothermal melting point apparatus and are uncorrected.

### Chemistry

**Scheme 1:**
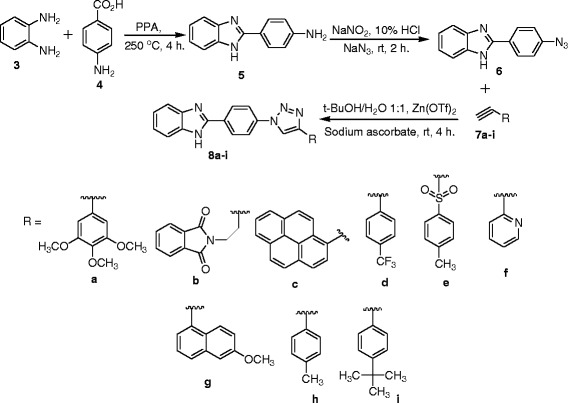
**Synthesis 1,2,3-triazoles.**

The synthesis of novel benzimidazole linked triazole (**8a-i**) derivatives is carried out as shown in Scheme 1. The key intermediate for the preparation of the new analogs is 2-(4-azidophenyl)-1H-benzo[d]imidazole (**6**). The mixture of O-phenylenediamine (**3**) and 4-aminobenzoic acid (**4**) was mixed with a sufficient quantity of polyphosphoric acid. The resulting solution was stirred at 250°C for 4 h, to afford compound **5**. Compound **5** was diazotizated followed by azidation to afford compound **6**. Compound **6** upon treatment with different types of terminal alkynes in t-BuOH/H_2_O, sodium ascorbate, and Zn(OTf)_2_ afforded compounds (**8a-i**).

### **4-(1H-Benzo[d]imidazol-2-yl)benzenamine**(**5**)

A mixture of the O-phenylenediamine (**3**) (500 mg, 3.64 mmol) and the 4-aminobenzoic acid (**4**) (394 mg, 3.64 mmol) was dissolved in sufficient quantity of polyphosphoric acid (PPA). The mixture was heated slowly to 250°C for 4 h, permitted to cool to room temperature, quenched with excess of 10% Na_2_CO_3_ solution, and extracted with ethyl acetate. Then, the mixture was dried over anhydrous Na_2_SO_4_, and the crude product was purified by column chromatography with ethyl acetate/hexane (6:4) to afford pure compound **5**, 946 mg in 97% yield. Mp: 209°C to 211°C, ^1^H NMR (300 MHz, DMSO-d_6_): *δ* 6.68 (d, 2H, *J* =7.3 Hz), 7.14 (br s, 2H), 7.50 (br s, 2H), 7.85 (d, 2H, *J* =7.1 Hz). IR (neat, cm^−1^): *γ*_max_ 404.3; 501.4; 537.9; 607.7; 742.3; 833.4; 960.1; 1,009.1; 1,108.6; 1,178.6; 1,225.7; 1,272.2; 1,397.2; 1,444.1; 1,499.2; 1,612.4; 1,701.4; 2,750.1; 2,853.1; 2,921.8; 3,355.4; 3,435.0; MS (ESI): 210 [M + H]^+^.

### **2-(4-Azidophenyl)-1H-benzo[d]imidazole**(**6**)

The amine derivative (**5**) (500 mg, 2.39 mmol) was dissolved in 10% aq HCl at room temperature. This reaction mixture upon cooling to 0°C and addition of a solution of NaNO_2_ (165 mg, 2.39 mmol) was stirred for 10 min at 0°C to 5°C. Sodium azide (186 mg, 2.87 mmol) was added, and the mixture was stirred at room temperature for 2 h. The reaction was worked up by dilution with ethyl acetate. The organic layer was washed with brine and dried over Na_2_SO_4_. After evaporation of the solvent, the crude product was purified by column chromatography with ethyl acetate/hexane (3:7) to afford pure compound **6**, 536 mg in 95% yield; Mp: 317°C to 319°C, ^1^H NMR (300 MHz, DMSO-d_6_): *δ* 7.16 to 7.26 (m, 2H), 7.32 (d, 2H, *J* =9.0 Hz), 7.50 to 7.69 (dd, 2H, *J* =40.0, 38.5 Hz), 8.22 (d, 2H, *J* =8.3 Hz), 12.97 (s, 1H). IR (neat, cm^−1^): *γ*_max_ 500.6; 541.4; 694.3; 743.3; 838.1; 963.4; 1,011.1; 1,115.8; 1,175.5; 1,283.5; 1,395.2; 1,438.8; 1,485.1; 1,604.6; 1,726.8; 2,120.1; 2,414.9; 2,856.9; 2,918.2; 3,055.5; 3,422.9; MS (ESI): 236 [M + H]^+^.

### 2-(4-(4-(3,4,5-Trimethoxyphenyl)-1H-1,2,3-triazol-1-yl)phenyl)-1H-benzo[d]imidazole (**8a**)

A mixture of the corresponding azide **6** (200 mg, 0.85 mmol) and the corresponding alkyne **7a** (163 mg, 0.85 mmol) was dissolved in t-BuOH/H_2_O 1:1 (20 mL). Sodium ascorbate (33 mg, 20 mol%) and Zn(OTf)_2_ (300 mg, 5 mol%) were added. After stirring for 4 h, water/ice (40 mL) was added. The product was either worked up by filtration, followed by rinsing with aqueous 5% NH_3_ (×3) and cold ether (×2), or by extraction with dichloromethane (4 × 100 mL). The combined organic layers were washed with aqueous 5% NH_3_ (3 × 100 mL) and brine (100 mL) and dried over anhydrous MgSO_4_. The solvent was removed *in vacuo*, and the crude product was purified by column chromatography with ethyl acetate/hexane (3:7) to afford pure compound **8a**, 347 mg in 95% yield. Mp: 270°C to 272°C, ^1^H NMR (400 MHz, CDCl_3_): *δ* 3.71 (s, 3H), 3.89 (s, 6H), 7.24 to 7.28 (m, 4H), 7.63 to 7.66 (m, 2H), 8.17 (d, 2H, *J* =8.4 Hz), 8.41 (d, 2H, *J* =8.4 Hz), 9.44 (s, 1H). ^13^C NMR (100 MHz, CDCl_3_): *δ* 57.2, 61.4, 110.2, 114.8, 117.1, 122.1, 123.3, 125.3, 128.4, 129.8, 140.1, 141.8, 143.3, 148.7, 152.4, 154.1; MS (ESI): 428 [M + H]^+^.

The other derivatives are also prepared according to the same procedure and described in Additional file 1.

### Biological evaluation

#### In vitro *cytotoxicity assay*

The synthesized compounds **8a-i** were evaluated for their anticancer activity in selected human cancer cell lines of A375, B-16, colon-205, MCF-7, and A-549 by using MTT assay. All the compounds (**8a-i**) exhibited significant anticancer activity with IC_50_ values ranging from 0.1 to 43 μM, while the positive control, adriamycin, demonstrated the IC_50_ in the range of 0.03 to 3.5 μM respectively, in the cell lines employed as shown in Table 1.

**Table 1:** **Cytotoxic activity (IC**_**50**_ **μM) of compounds 8a-i**

Compound	A375	B-16	Colon-205	MCF-7	A-549
**8a**	2.7	-	1.3	3.2	0.1
**8b**	2.3	1.9	3.5	1.7	2.6
**8c**	1.6	16	0.12	1.9	-
**8d**	-	4.7	8	-	-
**8e**	2.1	-	1.3	0.3	1.8
**8f**	15	3.9	17	-	-
**8g**	43	-	32	-	26
**8h**	-	-	-	37	32
**8i**	27	10	21	-	-
**ADR**	0.9	1.0	3.5	3.2	0.03

### Procedure for MTT assay

Toxicity of test compound in cells was determined by MTT assay based on mitochondrial reduction of yellow MTT tetrazolium dye to a highly colored blue formazan product. Cells (1 × 10^4^) (counted by Trypan blue exclusion dye method) in 96-well plates were incubated with compounds with series of concentrations tested for 48 h at 37°C in RPMI/DMEM/MEM with 10% FBS medium. Then, the above media was replaced with 90 μl of fresh serum free media and 10 μl of MTT reagent (5 mg/ml), and plates were incubated at 37°C for 4 h, thereafter the above media was replaced with 200 μl of DMSO and incubated at 37°C for 10 min. The absorbance at 570 nm was measured on a spectrophotometer (SpectraMax, Molecular devices, Sunnyvale, CA, USA). IC_50_ values were determined from plot: percent inhibition (from control) versus concentration.

## Additional file

## Electronic supplementary material

Additional file 1: Experimental procedure and characterization data of all new compounds. (DOC 2 MB)

Below are the links to the authors’ original submitted files for images.

Authors’ original file for figure 1

Authors’ original file for figure 2
