# Supplementary material for: Synthesis and biological evaluation of benzimidazole-linked 1,2,3-triazole congeners as agents
Source: Org Med Chem Lett. 2014 Dec 2;4:14. doi: 10.1186/s13588-014-0014-x (PMC4970438; doi:10.1186/s13588-014-0014-x)

**Synthesis and biological evaluation of benzimidazole Linked 1,2,3-triazole congeners as agents**

**Karna Ji Harkala^a^, Laxminarayana Eppakayala^a^*and Thirumala Chary^b^,**

**Supporting Information**

**Experimental section**

All chemicals and reagents were obtained from Aldrich (Sigma–Aldrich, St. Louis, MO, USA), Lancaster (Alfa Aesar, Johnson Matthey Company, Ward Hill, MA, USA) and were used without further purification. Reactions were monitored by TLC, performed on silica gel glass plates containing 60 F-254, and visualization on TLC was achieved by UV light or iodine indicator. ^1^H and ^13^C NMR spectra were recorded on Gemini Varian-VXR-unity (400 MHz) instrument. Chemical shifts (d) are reported in ppm downfield from internal TMS standard. ESI spectra were recorded on Micro mass, Quattro LC using ESI+ software with capillary voltage 3.98 kV and ESI mode positive ion trap detector. Melting points were determined with an electrothermal melting point apparatus, and are uncorrected.

**2-(4-(4-(3,4,5-Trimethoxyphenyl)-1H-1,2,3-triazol-1-yl)phenyl)-1H-benzo[d]imidazole** (**8a**). A mixture of the corresponding azide **6** (200 mg, 0.85 mmol) and the corresponding alkyne **7a** (163 mg, 0.85 mmol) was dissolved in t-BuOH/H_2_O 1:1 (20 mL). Sodium ascorbate (33 mg, 20 mol %) and Zn(OTf)_2_ (300 mg, 5 mol%) were added. After stirring 4 hours, water/ice (40 mL) was added. The product was either worked up by filtration, followed by rinsing with aqueous 5% NH_3_ (×3) and cold ether (×2), or by extraction with dichloromethane (4 × 100 mL). The combined organic layers were washed with aqueous 5% NH_3_ (3 × 100 mL) and brine (100 mL), and dried over anhydrous MgSO_4_. The solvent was removed in vacuo and the crude product was purified by column chromatography with ethyl acetate/hexane (3:7) to afford pure compound **8a**, 347 mg in 95% yield. Mp: 270–272 ^o^C, ^1^H NMR (400 MHz, CDCl_3_): *δ* 3.71 (s, 3H), 3.89 (s, 6H), 7.24−7.28 (m, 4H), 7.63−7.66 (m, 2H), 8.17 (d, 2H, *J* = 8.4 Hz), 8.41 (d, 2H, *J* = 8.4 Hz), 9.44 (s, 1H). ^13^C NMR (100 MHz, CDCl_3_): *δ* 57.2, 61.4, 110.2, 114.8, 117.1, 122.1, 123.3, 125.3, 128.4, 129.8, 140.1, 141.8, 143.3, 148.7, 152.4, 154.1; MS (ESI): 428 [M+H]^+^.

**2-(2-(1-(4-(1H-benzo[d]imidazol-2-yl)phenyl)-1H-1,2,3-triazol-4-yl)ethyl)isoindoline-1,3-dione** (**8b**). The compound **8b** was prepared following the method described for the preparation of the compound **8a**, employing **6** ( 200 mg, 0.85 mmol) and **7b** (169 mg, 0.85 mmol), and the crude product was purified by column chromatography with ethyl acetate/hexane (2:8) to afford pure compound **8b**, 359 mg in 97% yield. Mp: 282–284 ^o^C, ^1^H NMR (400 MHz, CDCl_3_): *δ* 3.10 (t, 2H, *J* = 6.9, 6.9 Hz), 3.91 (t, 2H, *J* = 6.9, 6.9 Hz), 7.20−7.29 (m, 2H), 7.59−7.67 (m, 2H), 7.81−7.91 (m, 4H), 8.07 (d, 2H, *J* = 8.4 Hz), 8.36 (d, 2H, *J* = 8.4 Hz), 8.82 (s, 1H). ^13^C NMR (100 MHz, CDCl_3_): *δ* 23.9, 28.2, 114.9, 115.8, 122.8, 123.0, 123.6, 125.0, 128.9, 129.1, 134.1, 140.1, 141.6, 142.3, 151.9, 172.3; MS (ESI): 435 [M+H]^+^.

**2-(4-(4-(pyren-2-yl)-1H-1,2,3-triazol-1-yl)phenyl)-1H-benzo[d]imidazole (8c).**

The compound **8c** was prepared following the method described for the preparation of the compound **8a**, employing **6** (200 mg, 0.85 mmol) and **7c** (192 mg, 0.85 mmol), and the crude product was purified by column chromatography with ethyl acetate/hexane (1:1) to afford pure compound **8c**, 298 mg in 76% yield. Mp: 264–266 ^o^C, ^1^H NMR (500 MHz, CDCl_3_): *δ* 3.65 (m, 2H), 6.21-6.38 (m, 3H), 6.49 (d, 1H, *J* = 8.3 Hz), 6.89 (s, 2H), 7.24-7.30 (m, 4H), 7.49-7.50 (m, 3H), 7.69 (d, 2H, *J* = 8.5 Hz), 7.89 (s, 1H). ^13^C NMR (100 MHz, CDCl_3_): *δ* 30.9, 36.2, 52.5, 57.8, 114.1, 116.1, 123.1, 123.3, 125.1, 129.2, 134.1, 134.8, 139.7, 147.9, 152.1. MS (ESI): 464 [M+H]^+^.

**2-(4-(4-(4-(Trifluoromethyl)phenyl)-1H-1,2,3-triazol-1-yl)phenyl)-1H-benzo[d]imidazole** (**8d**). The compound **8d** was prepared following the method described for the preparation of the compound **8a**, employing **6** (200 mg, 0.85 mmol) and **7d** (144 mg, 0.85 mmol), and the crude product was purified by column chromatography ethyl acetate/hexane (3:7) to afford the compound **8d**, 339 mg in 98% yield. Mp: 316–318 ^o^C, ^1^H NMR (300 MHz, CDCl_3_): *δ* 7.19−7.27 (m, 3H), 7.53−7.85 (m, 5H), 8.17 (d, 2H, *J* = 9.0 Hz), 8.41 (d, 2H, *J* = 8.3 Hz), 9.50 (s, 1H). ^13^C NMR (100 MHz, CDCl_3_): *δ* 112.9, 115.4, 122.1, 123.2, 123.8, 126.1, 126.3, 127.3, 130.1, 130.3, 133.1, 140.2, 142.3, 147.4, 152.8; MS (ESI): 406 [M+H]^+^.

**2-(4-(4-Tosyl-1H-1,2,3-triazol-1-yl)phenyl)-1H-benzo[d]imidazole** (**8e**)**.**

The compound **8e** was prepared following the method described for the preparation of the compound **8a**, employing **6** (200 mg, 0.85 mmol) and **7e** (153 mg, 0.85 mmol), and the crude product was purified by column chromatography with ethyl acetate/hexane (3:7) to afford pure compound **8e**, 347 mg in 98% yield. Mp: 222–224 ^o^C, ^1^H NMR (300 MHz, CDCl_3_): *δ* 2.41 (s, 3H), 7.18−7.30 (m, 2H), 7.50 (d, 2H, *J* = 8.1 Hz), 7.55−7.74 (dd, 2H), 7.94 (d, 2H, *J* = 8.1 Hz), 8.16 (d, 2H, *J* = 8.4 Hz), 8.39 (d, 2H, *J* = 8.4 Hz), 9.78 (s, 1H). ^13^C NMR (100 MHz, CDCl_3_): *δ* 21.5, 112.4, 115.9, 122.1, 123.3, 127.4, 127.8, 130.1, 131.3, 139.8, 144.1, 147.8, 150.9, 153.2; MS (ESI): 416 [M+H]^+^.

**2-(4-(4-(Pyridin-2-yl)-1H-1,2,3-triazol-1-yl)phenyl)-1H-benzo[d]imidazole** (**8f**).

The compound **8f** was prepared following the method described for the preparation of the compound **8a**, employing **6** (200 mg, 0.85 mmol) and **7f** (87 mg, 0.85 mmol), and the crude product was purified by column chromatography with ethyl acetate/hexane (3:7) to afford pure compound **8f**, 274 mg in 95% yield. Mp: 338–340 ^o^C, ^1^H NMR (300 MHz, CDCl_3_): *δ* 7.19 (br s, 2H), 7.42–7.63 (m, 3H), 8.11 (d, 2H, *J* = 8.3 Hz), 8.28–8.31 (m, 1H), 8.42 (d, 2H, *J* = 8.3 Hz), 8.49–8.61 (m, 1H), 9.16 (s, 1H), 9.45 (s, 1H). ^13^C NMR (100 MHz, CDCl_3_): *δ* 115.9, 121.1, 122.7, 122.9, 123.1, 123.9, 126.1, 130.3, 135.8, 140.1, 141.2, 141.4, 150.3, 153.2, 153.4; MS (ESI): 339 [M+H]^+^.

**2-(4-(4-(6-Methoxynaphthalen-1-yl)-1H-1,2,3-triazol-1-yl)phenyl)-1H-benzo[d]imidazole** (**8g**). The compound **8g** was prepared following the method described for the preparation of the compound **8a**, employing **6** (200 mg, 0.85 mmol) and **7g** (154 mg, 0.85 mmol), and the crude product was purified by column chromatography with ethyl acetate/hexane (3:7) to afford pure compound **8g**, 348 mg in 98% yield. Mp: 310–314 ^o^C, ^1^H NMR (300 MHz, CDCl_3_): *δ* 4.04 (s, 3H), 7.30–7.56 (m, 5H), 7.62–7.86 (m, 4H), 8.02–8.22 (m, 2H), 8.35 (d, 1H, *J* = 7.5 Hz), 8.57 (d, 2H, *J* = 8.4 Hz), 9.64 (s, 1H). ^13^C NMR (100 MHz, CDCl_3_): *δ* 53.8, 101.9, 116.8, 117.2, 118.3, 120.9, 121.7, 123.4, 126.1, 126.8, 127.6, 128.9, 130.1, 132.2, 132.4, 136.8, 140.2, 144.8, 147.2, 154.2, 156.7; MS (ESI): 418 [M+H]^+^.

**2-(4-(4-p-tolyl-1H-1,2,3-triazol-1-yl)phenyl)-1H-benzo[d]imidazole (8h).**

The compound **8h** was prepared following the method described for the preparation of the compound **8a**, employing **6** (200 mg, 0.85 mmol) and **7h** (99.1 mg, 0.85 mmol), and the crude product was purified by column chromatography with ethyl acetate/hexane (3:7) to afford pure compound **8h**, 250 mg in 83% yield. Mp: 260–262 ^o^C, ^1^H NMR (500 MHz, CDCl_3_): *δ* 2.30, 7.20 (d, 2H, *J* = 7.9 Hz), 7.23-7.31 (m, 6H), 7.48 (d, 2H, *J* = 8.2 Hz), 7.69 (d, 2H, *J* = 8.0 Hz), 7.88 (s, 1H). ^13^C NMR (100 MHz, CDCl_3_): *δ* 21.0, 115.1, 116.0, 123.2, 125.2, 125.5, 127.1, 130.3, 130.6, 138.8, 140.2, 142.8, 145.8, 154.3; MS (ESI): 352 [M+H]^+^.

**2-(4-(4-(4-tert-butylphenyl)-1H-1,2,3-triazol-1-yl)phenyl)-1H-benzo[d]imidazole (8i).**

The compound **8i** was prepared following the method described for the preparation of the compound **8a**, employing **6** (200 mg, 0.85 mmol) and **7i** (0.16 ml, 0.85 mmol), and the crude product was purified by column chromatography with ethyl acetate/hexane (4:6) to afford pure compound **8i**, 280 mg in 83% yield. Mp: 259–261 ^o^C, ^1^H NMR (500 MHz, CDCl_3_): *δ* 1.29 (s, 9H), 7.19-7.31 (m, 8H), 7.47 (d, 2H), 7.69 (d, 2H), 7.89 (s, 1H). ^13^C NMR (100 MHz, CDCl_3_): *δ* 32.3, 35.6, 115.4, 117.1, 120.1, 124.1, 125.8, 126.3, 126.8, 127.8, 129.5, 140.0, 141.8, 144.4, 151.9, 153.4; MS (ESI): 394 [M+H]^+^.

**Procedure for MTT-Assay.**

Toxicity of test compound in cells was determined by MTT assay based on mitochondrial reduction of yellow MTT tetrazolium dye to a highly colored blue formazan product. 1x10^4^ Cells (counted by Trypan blue exclusion dye method) in 96-well plates were incubated with compounds with series of concentrations tested for 48 hrs at 37 ^o^C in RPMI/DMEM/MEM with 10% FBS medium. Then the above media was replaced with 90 µl of fresh serum free media and 10 µl of MTT reagent (5mg/ml) and plates were incubated at 37 ^o^C for 4 h, there after the above media was replaced with 200 µl of DMSO and incubated at 37 ^o^C for 10 min. The absorbance at 570 nm was measured on a spectrophotometer (spectra max, Molecular devices) IC_50_ values were determined from plot : % inhibition (from control) versus concentration.


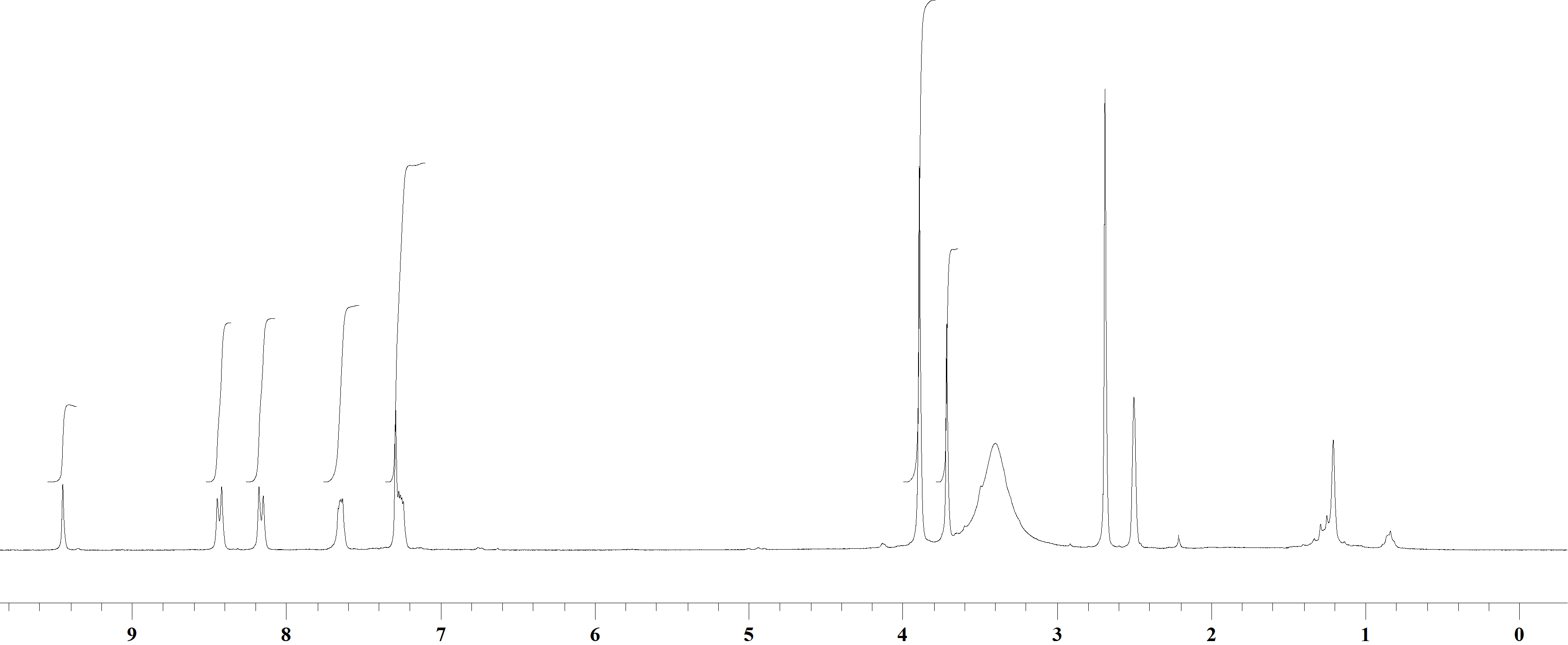


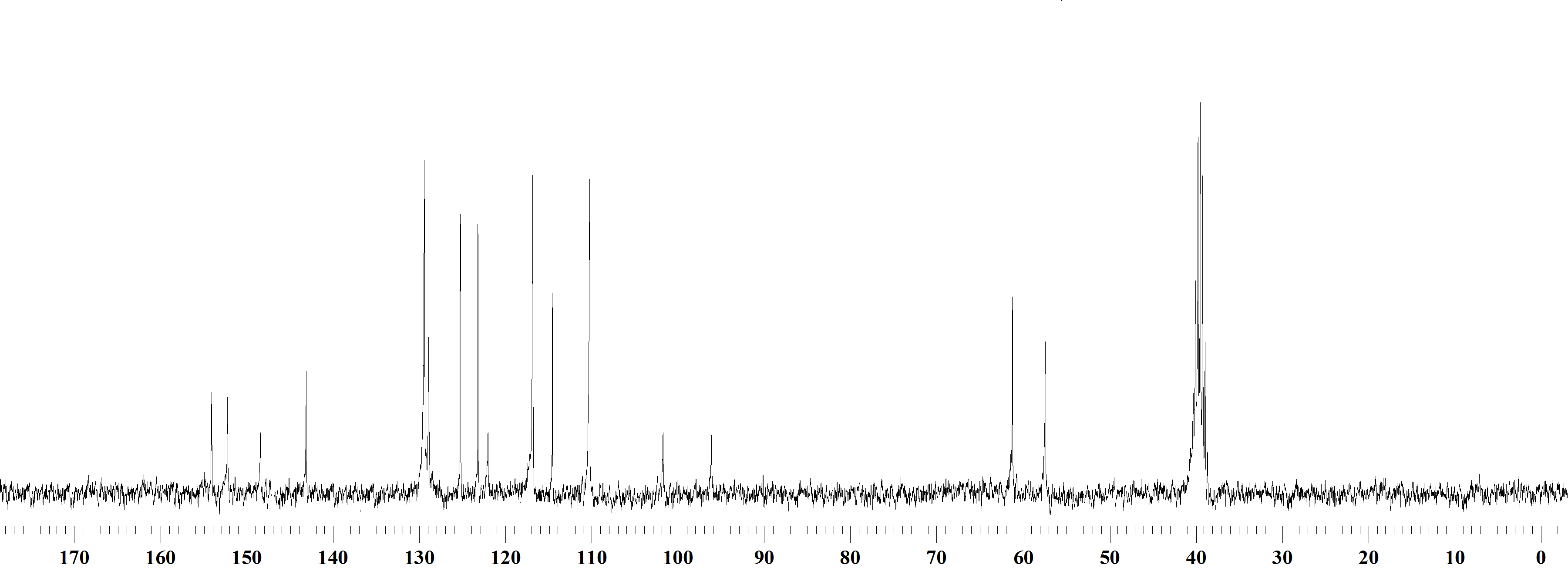


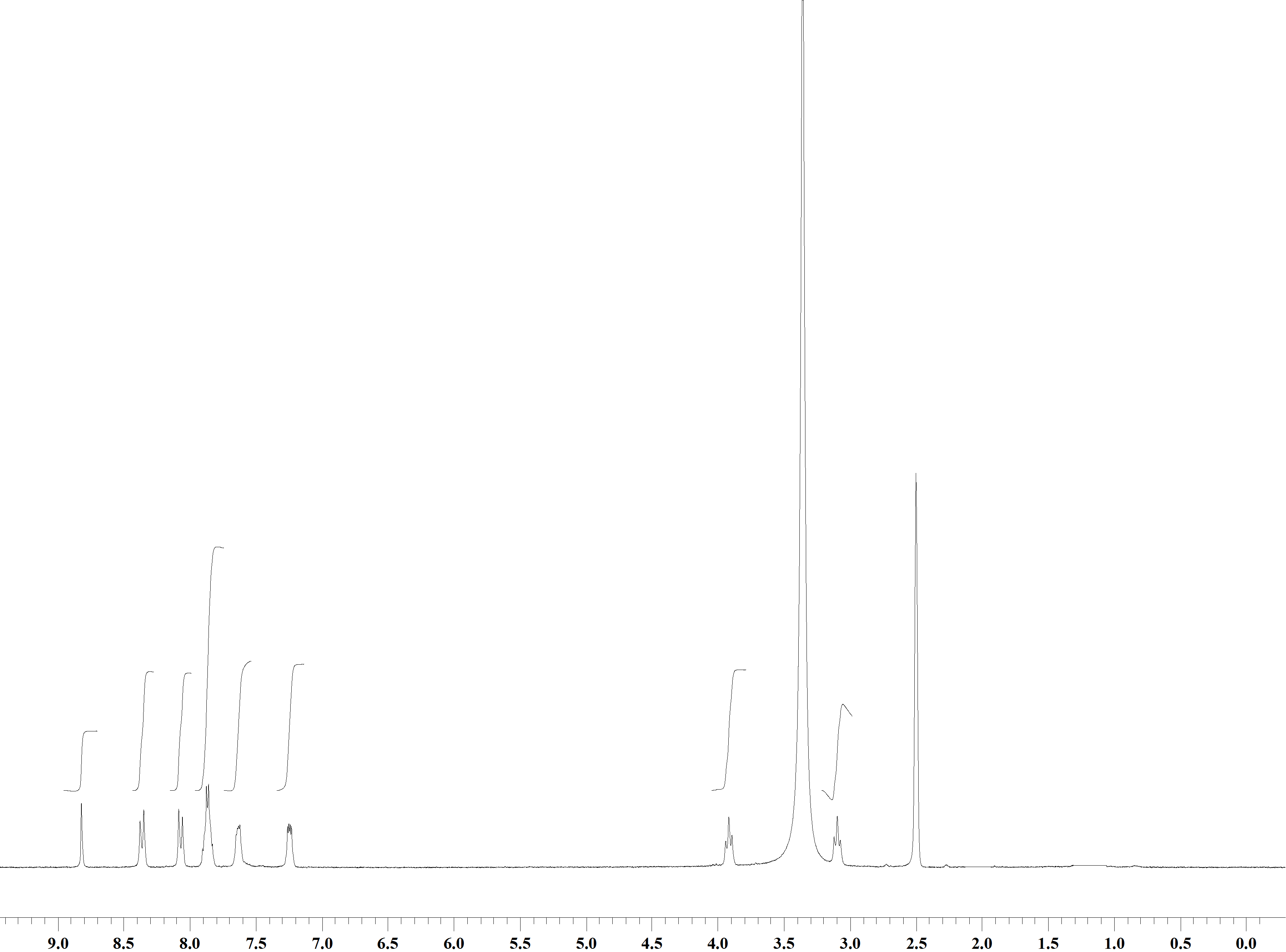


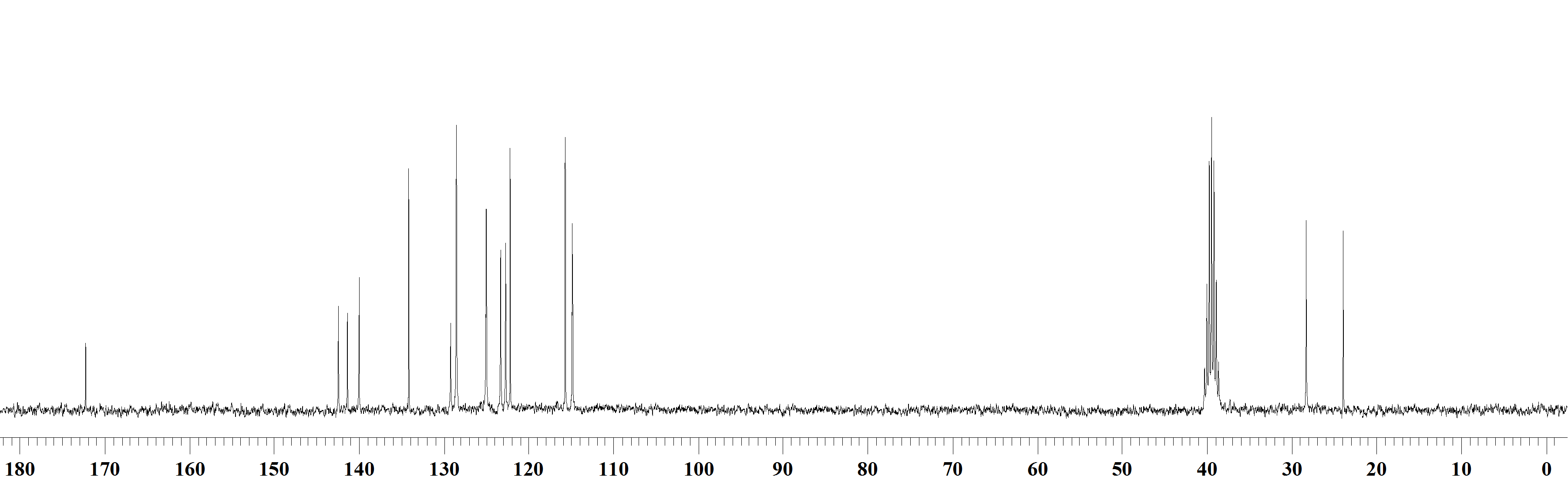


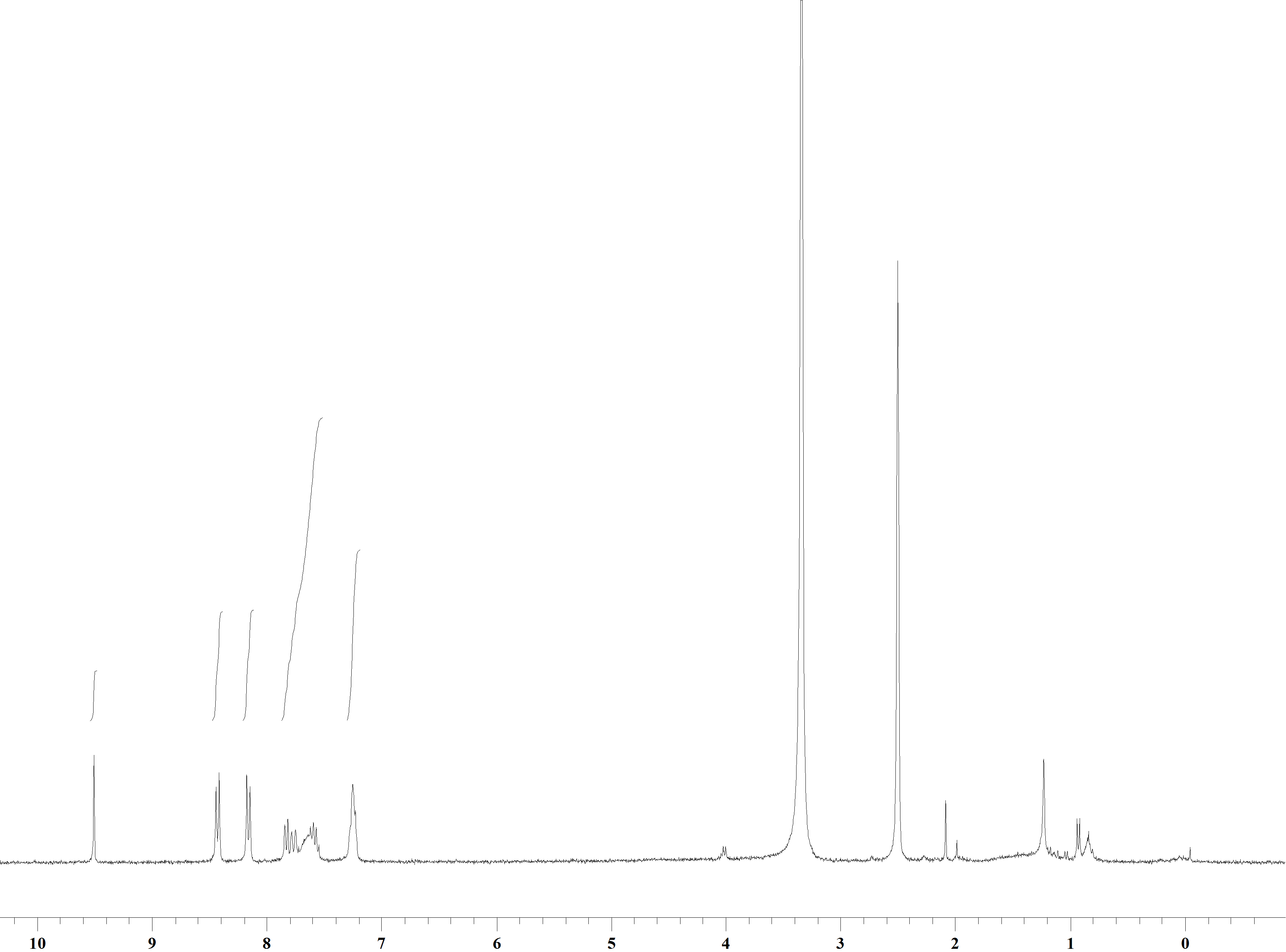


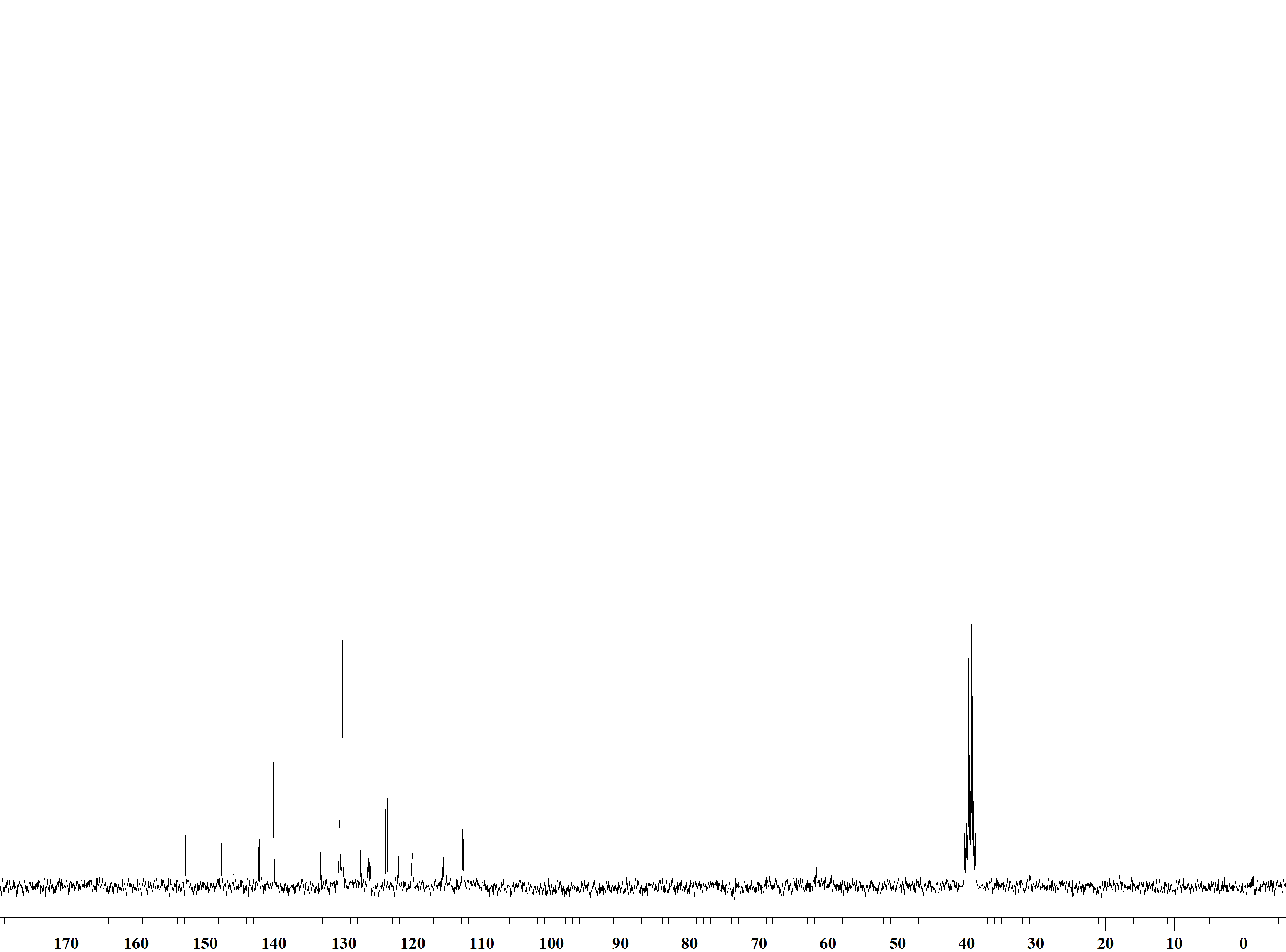


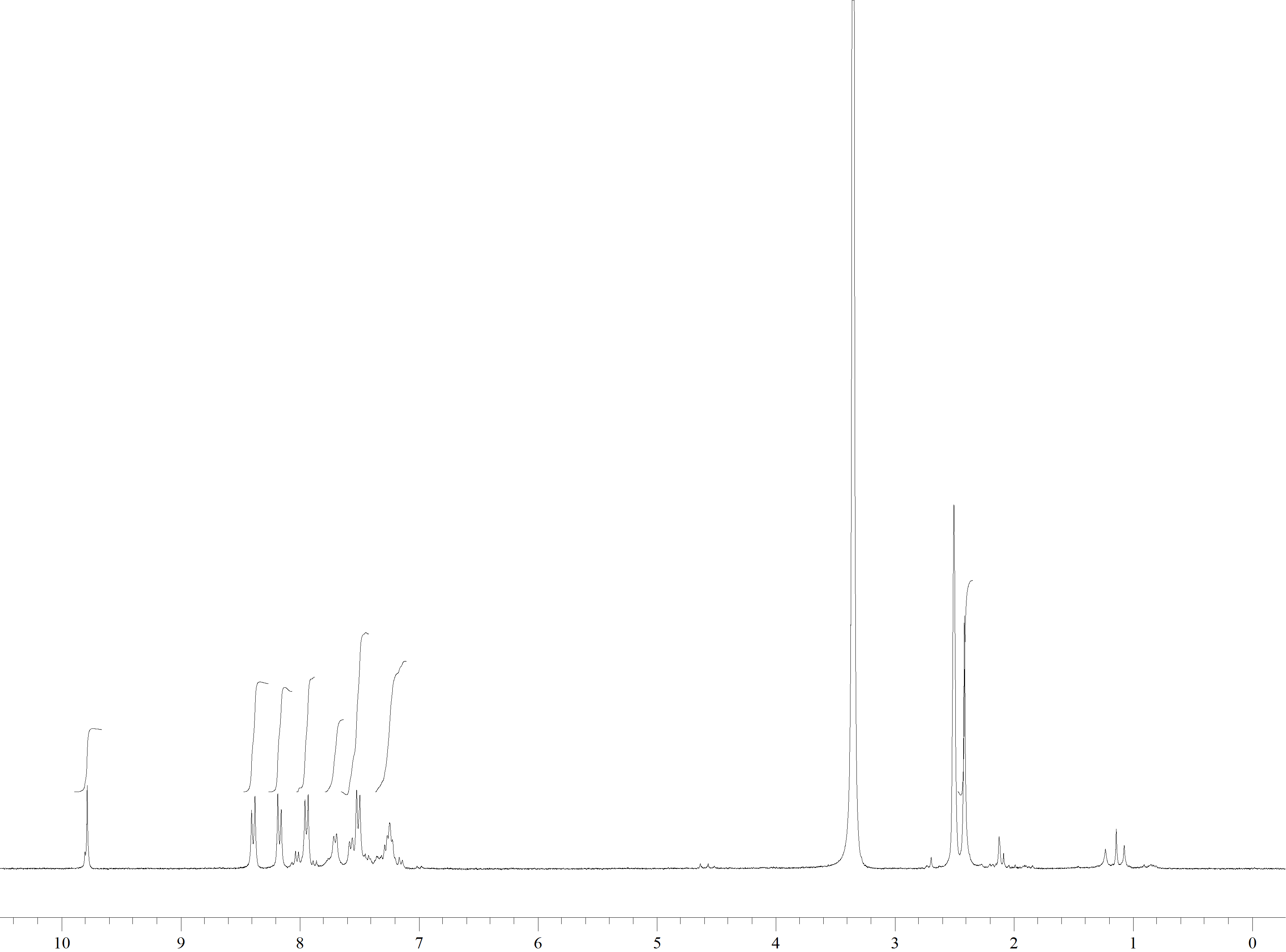


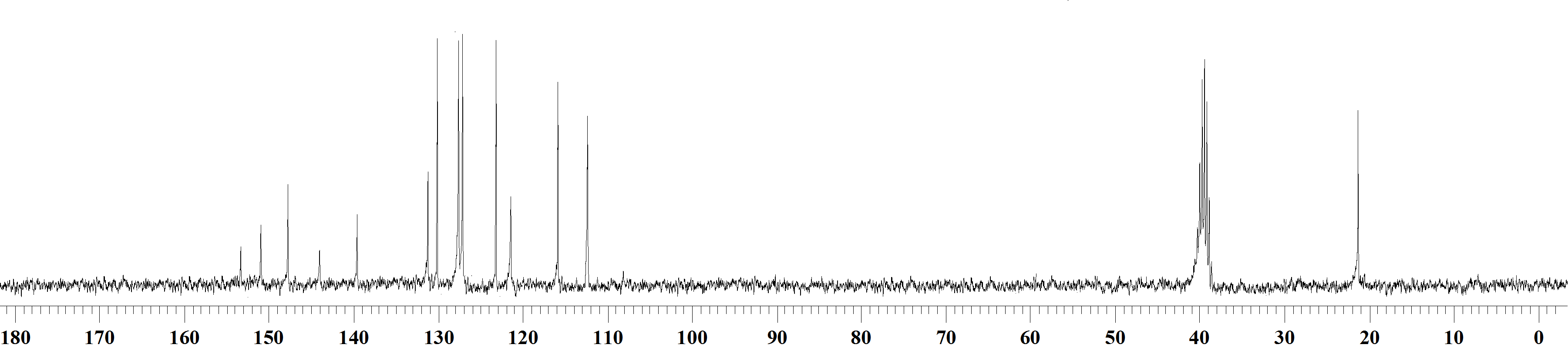


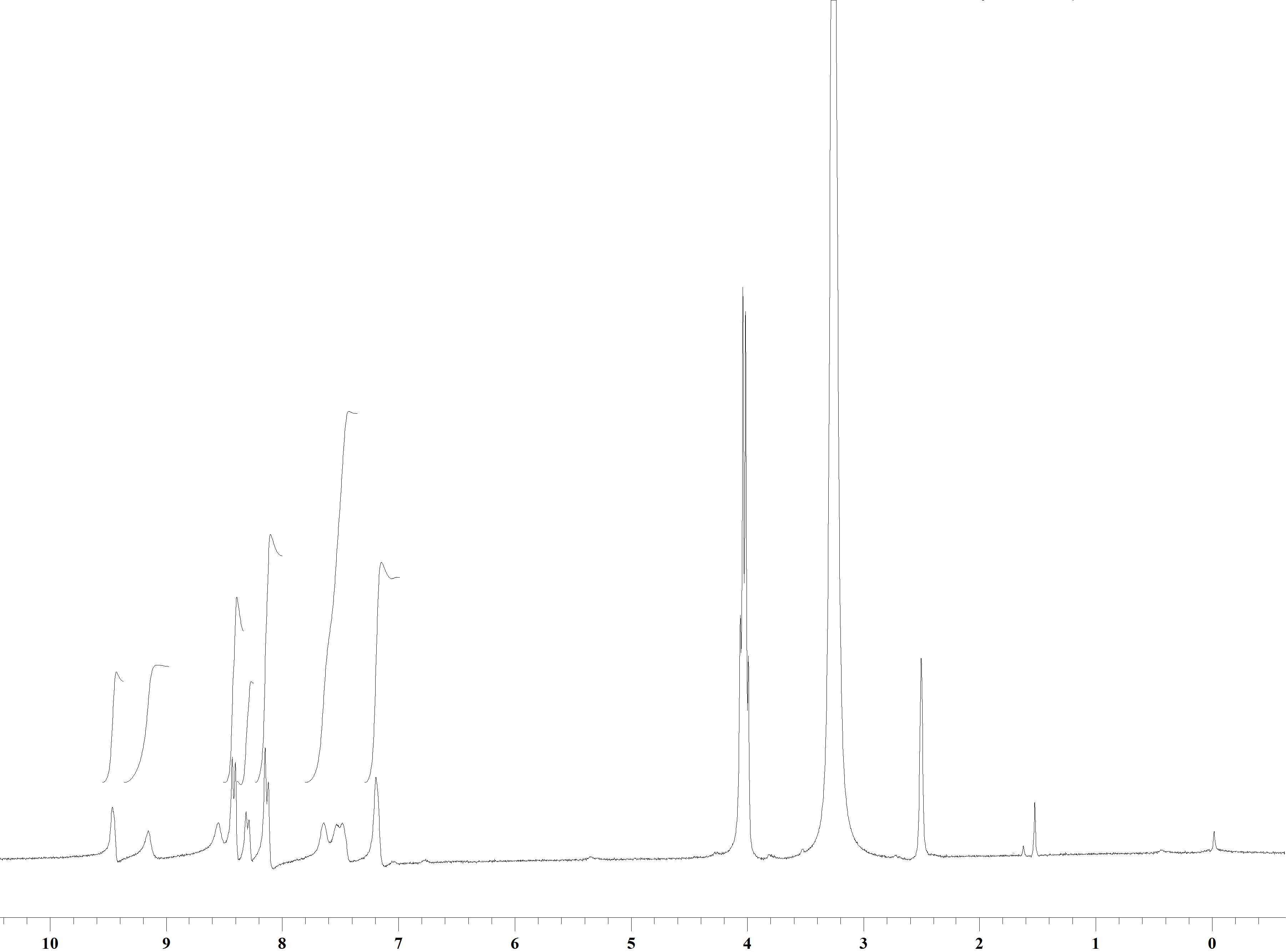


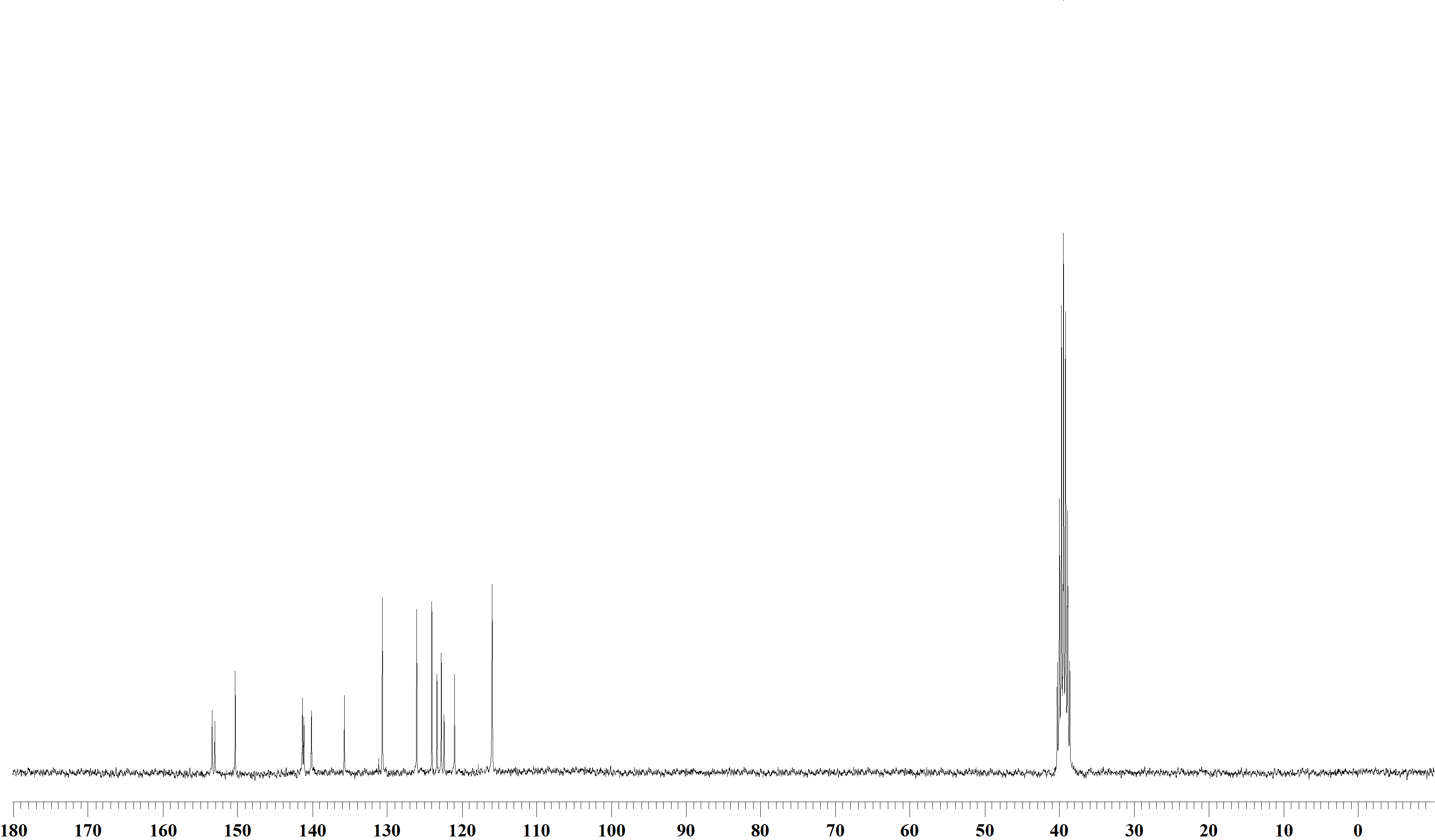


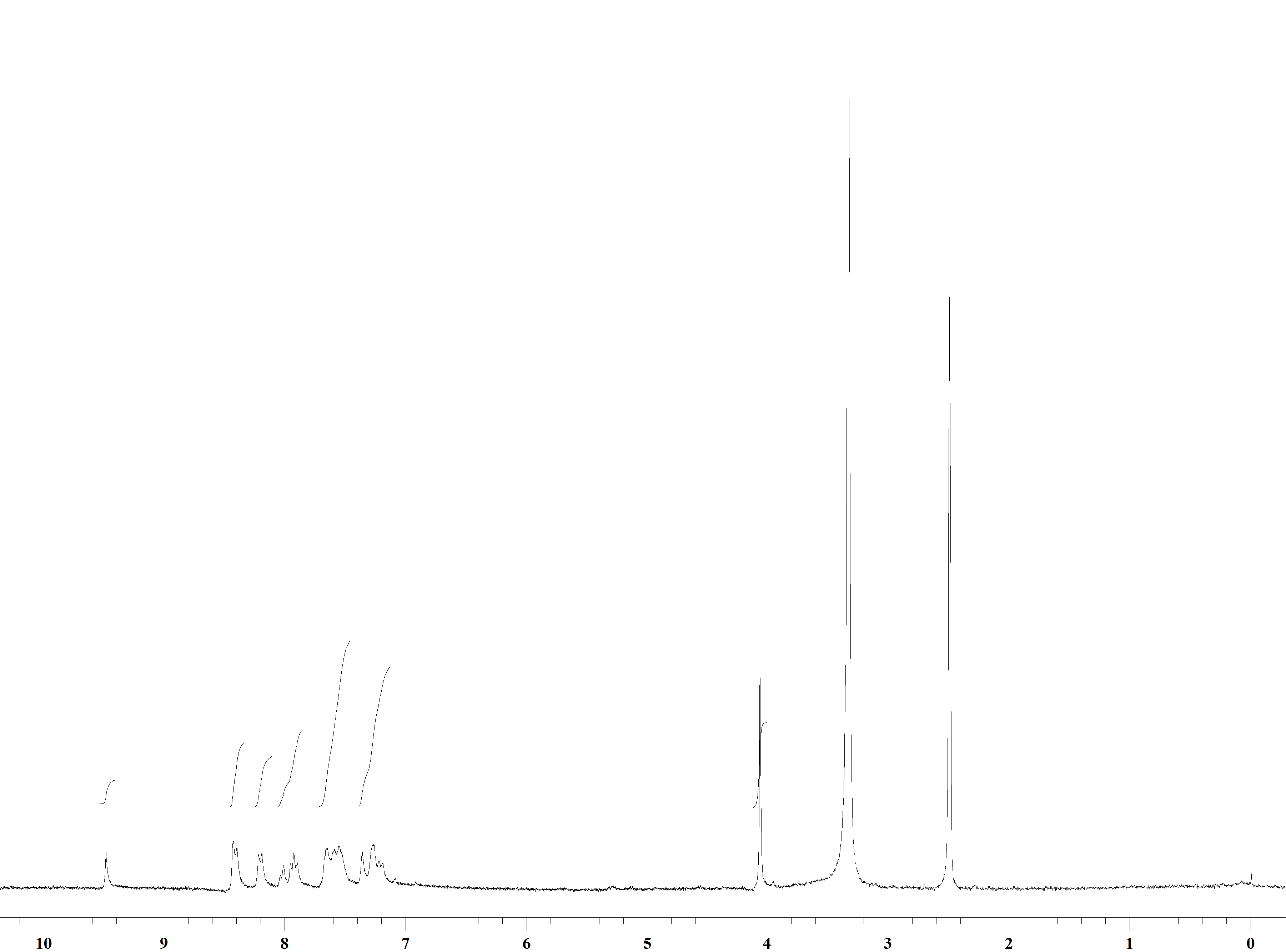


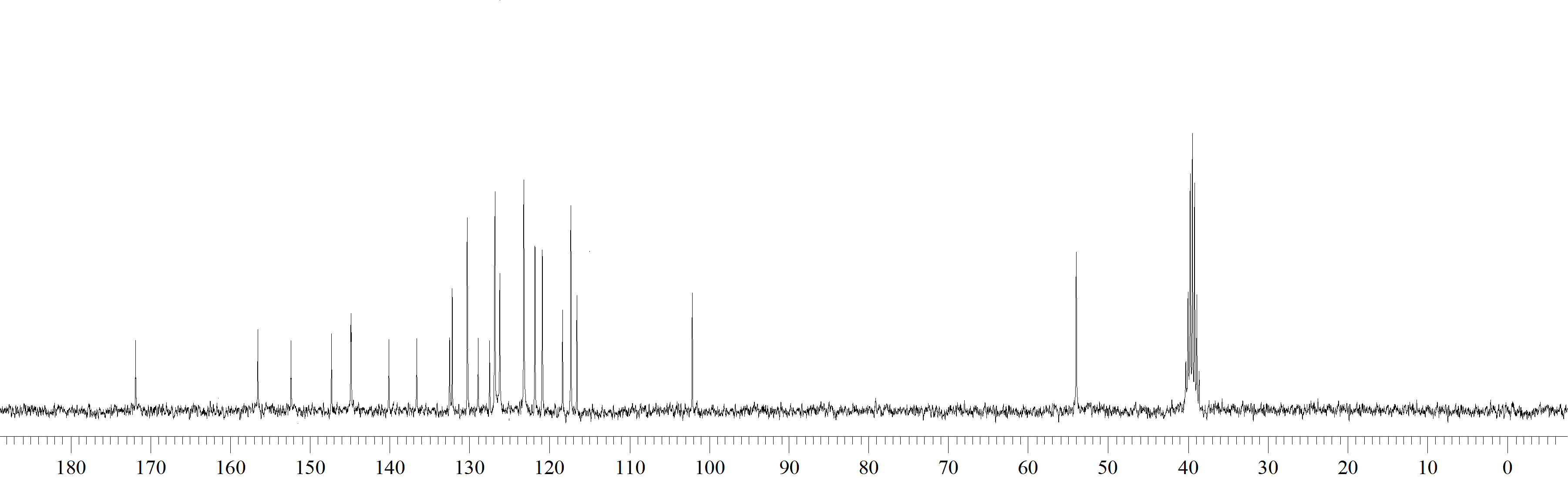


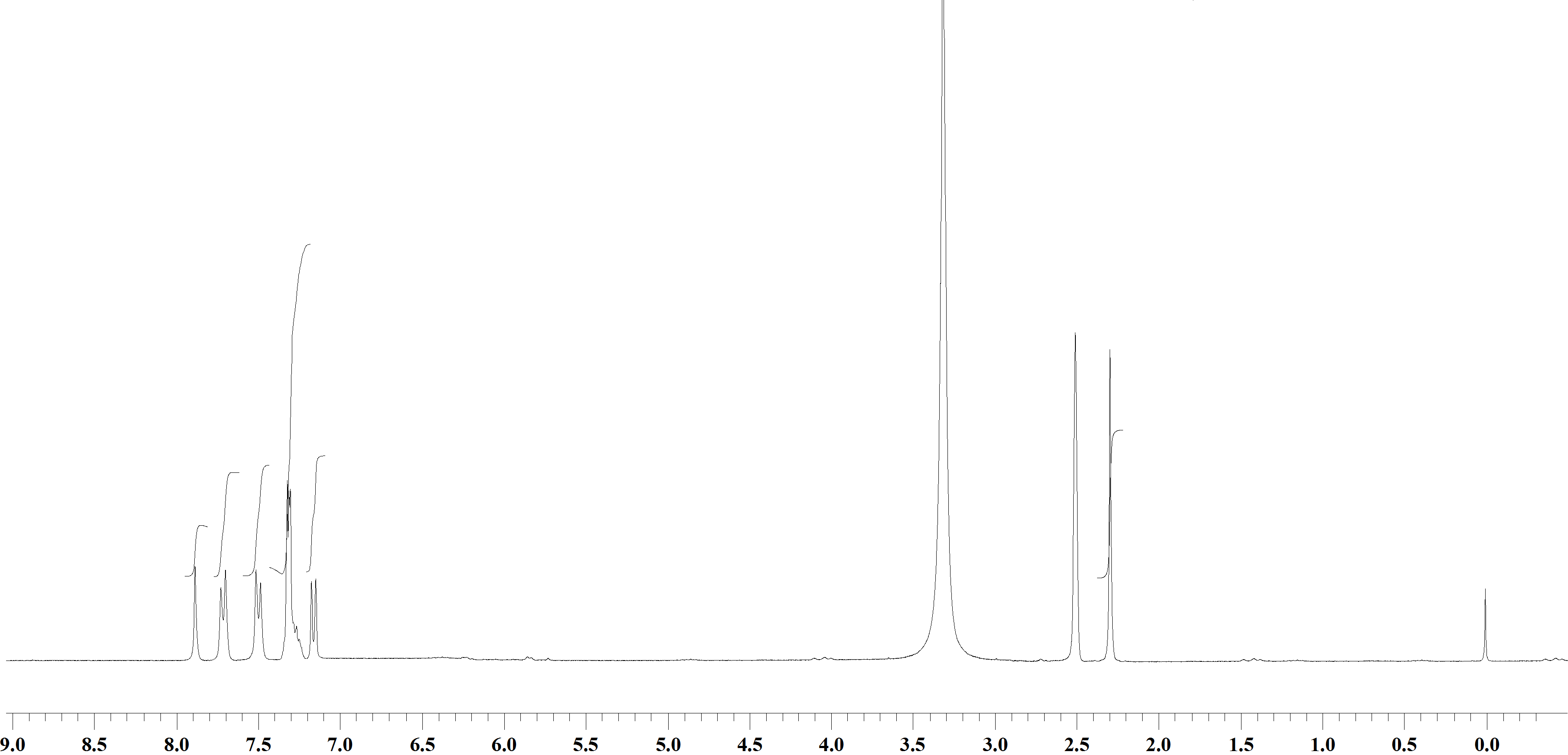


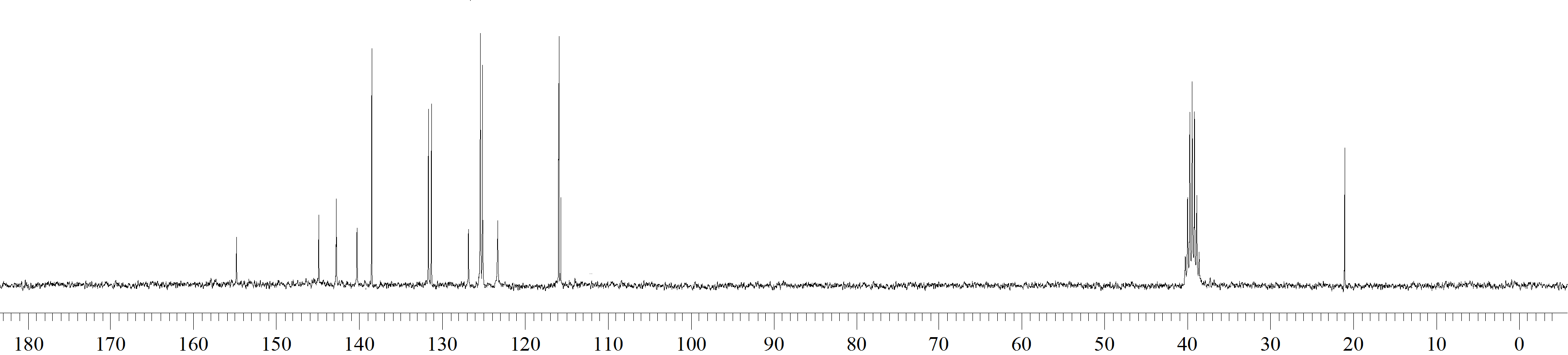


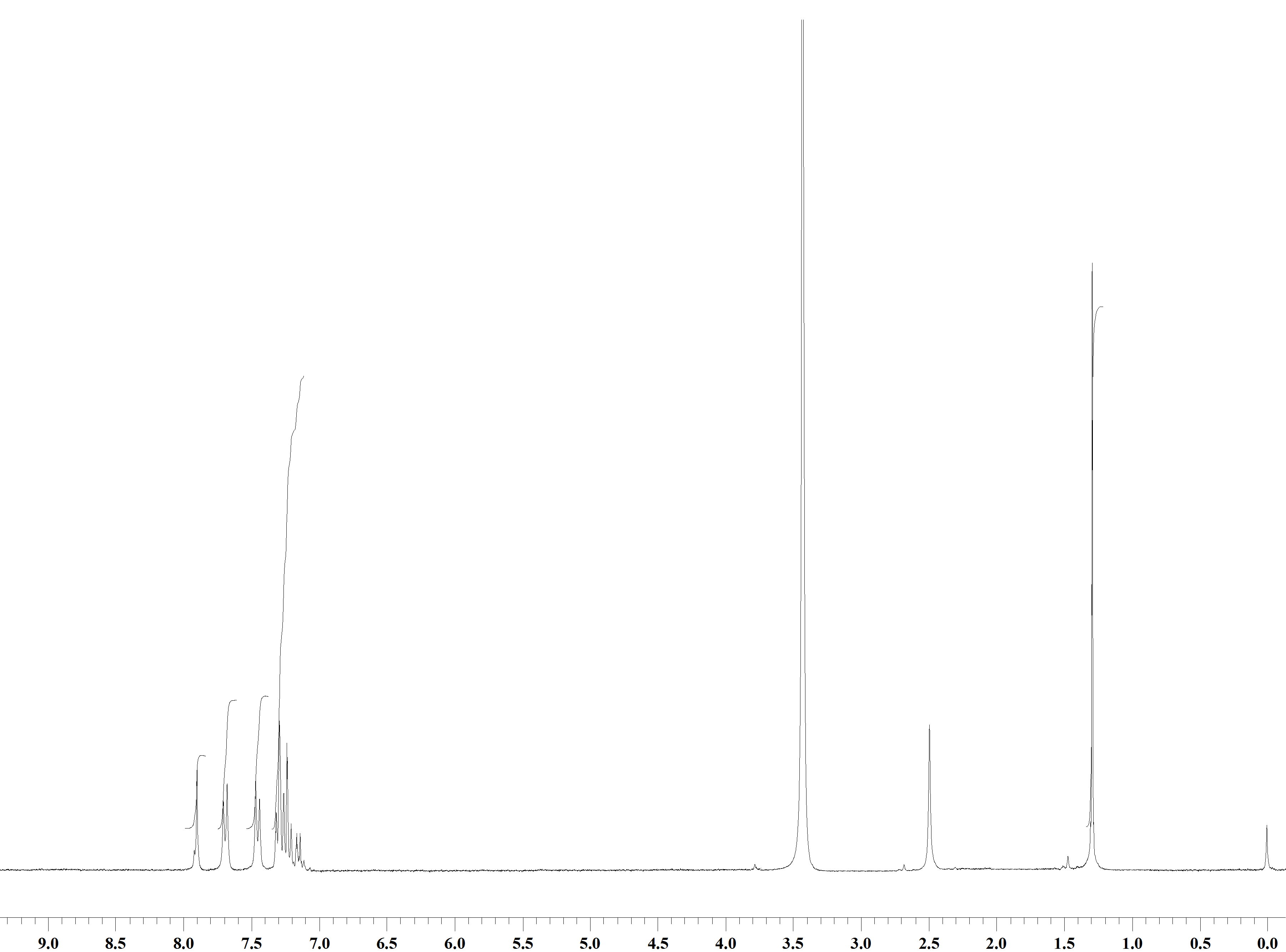


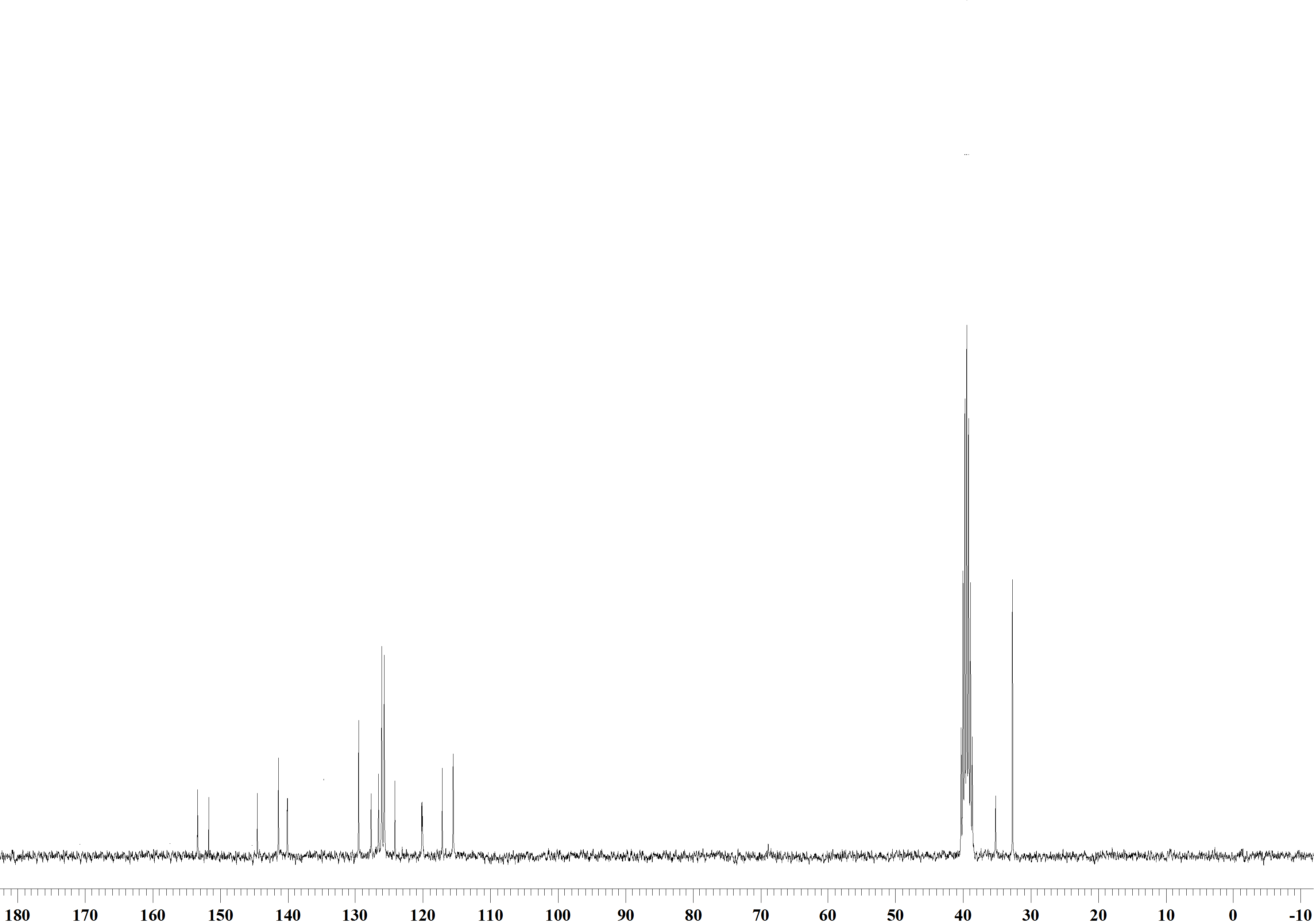

Supplement: Supplementary file 1 — Additional file 1: Experimental procedure and characterization data of all new compounds. (DOC 2 MB) [file 13588_2014_14_MOESM1_ESM.doc]
