# Supplementary figures and images for: Synthesis and biological evaluation of benzimidazole-linked 1,2,3-triazole congeners as agents
Source: Org Med Chem Lett. 2014 Dec 2;4:14. doi: 10.1186/s13588-014-0014-x (PMC4970438; doi:10.1186/s13588-014-0014-x)

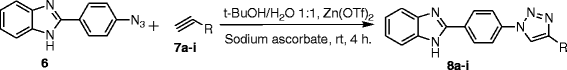

Supplement: Supplementary file 2 — Authors’ original file for figure 1 [file 13588_2014_14_MOESM2_ESM.gif]

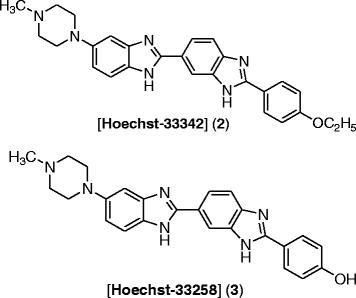

Supplement: Supplementary file 3 — Authors’ original file for figure 2 [file 13588_2014_14_MOESM3_ESM.gif]
